# Structural analysis of X-linked retinoschisis mutations reveals distinct classes which differentially effect retinoschisin function

**DOI:** 10.1093/hmg/ddw345

**Published:** 2016-10-23

**Authors:** Ewan P. Ramsay, Richard F. Collins, Thomas W. Owens, C. Alistair Siebert, Richard P.O. Jones, Tao Wang, Alan M. Roseman, Clair Baldock

**Affiliations:** 1 Wellcome Trust Centre for Cell-Matrix Research, School of Biological Sciences, Faculty of Biology, Medicine and Health, University of Manchester, Manchester, UK; 2 School of Biological Sciences, Faculty of Biology, Medicine and Health, University of Manchester, Manchester, UK; 3 Electron Bio-Imaging Centre, Diamond Light Source, Harwell Science and Innovation Research Campus, UK

## Abstract

Retinoschisin, an octameric retinal-specific protein, is essential for retinal architecture with mutations causing X-linked retinoschisis (XLRS), a monogenic form of macular degeneration. Most XLRS-associated mutations cause intracellular retention, however a subset are secreted as octamers and the cause of their pathology is ill-defined. Therefore, here we investigated the solution structure of the retinoschisin monomer and the impact of two XLRS-causing mutants using a combinatorial approach of biophysics and cryo-EM. The retinoschisin monomer has an elongated structure which persists in the octameric assembly. Retinoschisin forms a dimer of octamers with each octameric ring adopting a planar propeller structure. Comparison of the octamer with the hexadecamer structure indicated little conformational change in the retinoschisin octamer upon dimerization, suggesting that the octamer provides a stable interface for the construction of the hexadecamer. The H207Q XLRS-associated mutation was found in the interface between octamers and destabilized both monomeric and octameric retinoschisin. Octamer dimerization is consistent with the adhesive function of retinoschisin supporting interactions between retinal cell layers, so disassembly would prevent structural coupling between opposing membranes. In contrast, cryo-EM structural analysis of the R141H mutation at ∼4.2Å resolution was found to only cause a subtle conformational change in the propeller tips, potentially perturbing an interaction site. Together, these findings support distinct mechanisms of pathology for two classes of XLRS-associated mutations in the retinoschisin assembly.

## Introduction

The retina is a unique neural tissue, possessing pronounced laminar architecture with maintenance of retinal structure critical to neural processing (1). X-Linked Retinoschisis (XLRS) is a currently incurable, progressive condition that leads to juvenile-onset macular degeneration in males that results in loss of vision with splitting between inner nuclear layers and loss of normal retinal cytoarchitecture (2).

XLRS is caused by over 230 mutations in the RS1 gene, as reported by the HMGD Professional database (3), which encodes the protein retinoschisin (4,5). Retinoschisin, a 24kDa protein secreted by photoreceptors, consists of a retinoschisin (Rs1) domain and a discoidin domain with a small C-terminal extension (6–8). The discoidin domain is a conserved domain involved in adhesion interactions of many cell-matrix proteins that are thought to be mediated by three projecting loops or ‘spike’ regions (9). Uniquely, the cysteine rich Rs1 domain and the C-terminal extension in retinoschisin mediate the formation of a C59-C223 disulfide linkage which is essential for octamerisation (10). The resulting octameric complex is secreted and diffuses throughout the retina, attaching to the outer plasma membrane leaflet (11,12). This is crucial for maintenance of normal retinal cytoarchitecture. Deletion of retinoschisin in mouse models leads to the development of an XLRS-like phenotype (13) which is rescued by the introduction of wild-type retinoschisin (14–18). The majority of XLRS-associated mutations cause intracellular retention of retinoschisin (19–23). However, a subset of mutations (including R141H and H207Q) are still secreted as octamers (22). Despite observations that retinoschisin binds Na^+^/K^+^-ATPase (24) and L-type Voltage Gated Calcium ion Channels (L-VGCCs) (25), the molecular mechanism of retinoschisin function remains elusive.

Recently, the structure of octameric retinoschisin was determined using negative stain and cryo-EM showing assembly of the molecule into a hexadecameric structure of two octamers (26,27). However, the effects of secreted XLRS-associated mutations (in particular, R141H and H207Q) on this structure are unknown. Therefore, analysis of such mutations may prove crucial for elucidating the mechanism by which retinoschisin maintains retinal architecture.

In this study we analyse the assembly of retinoschisin through solution of the structure of the retinoschisin monomer coupled with cryo-EM analysis of the structure of an R141H XLRS mutant at 4.2Å resolution. Furthermore, an uncharacterized H207Q mutation was identified at the interface of the dimer of octamers and the effects of these two XLRS-associated mutations on the structure and stability of retinoschisin was investigated.

## Results

### The retinoschisin monomer has an elongated structure

**Figure 1.**
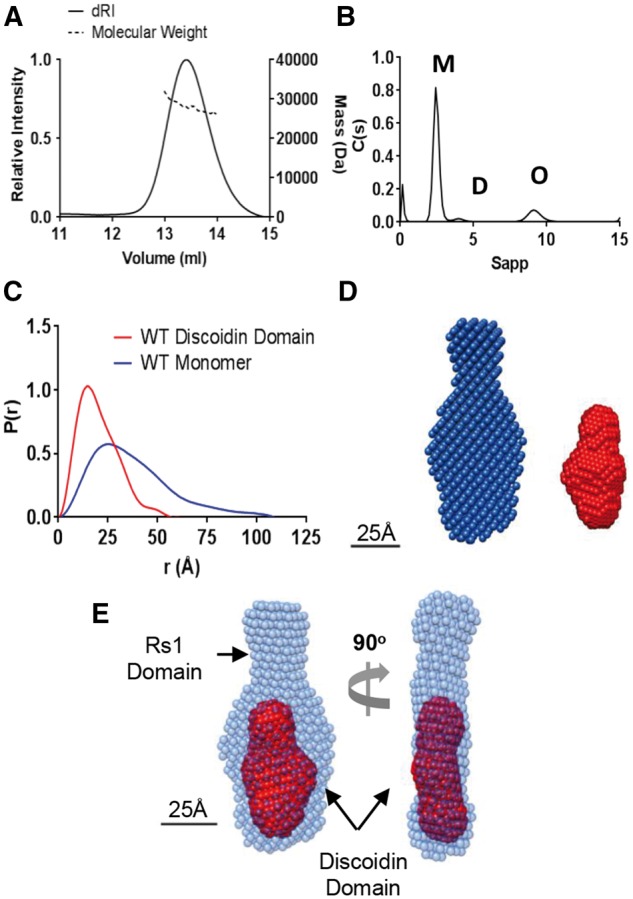
Structure of the retinoschisin monomer. (**A**) SEC-MALS analysis of wild-type retinoschisin monomer shows a molecular weight of approximately 27 kDa. (**B**) AUC analysis of monomeric (M) retinoschisin indicates an *S_20,w_* 2.6S, *R_h_* 2.4 nm and an *f/f_0_* 1.21, dimeric (D) and octameric (O) species are marked. (**C**) Overlayed pair distribution profiles from SAXS data for the retinoschisin monomer and discoidin domain with D_max_ of 10.8 nm and 5.5 nm for the monomer and isolated discoidin domain, respectively. (**D**) DAMMIF volumetric bead modelling of the retinoschisin monomer (blue) and the discoidin domain (red) modelled *ab initio* from the SAXS data. (**E**) Comparison of the retinoschisin monomer and discoidin domain revealing the relative positions of the two domains.

In order to determine the structure of the retinoschisin monomer, wild-type protein was expressed and purified from mammalian cells from a mixture of octameric, dimeric and monomeric species (Supplementary Material, Fig. S1). Multiangle Light Scattering (MALS) analysis of retinoschisin monomer revealed a molecular weight of approximately 27 kDa consistent with sequence predictions (Fig. 1A). Analytical ultracentrifugation (AUC) analysis provided a sedimentation coefficient (S_20,w_) of 2.6 S, a hydrodynamic radius (R_h_) of 2.4 nm and a *f/f_0_* value of 1.21 (Fig. 1B) indicating a globular structure. Small Angle X-Ray Scattering (SAXS) confirmed this elongated structure with the radius of gyration (R_g_) of 31.6 Å and maximum dimension (D_max_) of 108 Å (Figs. 1C and D and Supplementary Material, Fig. S2). Retinoschisin was further probed through SAXS analysis of the discoidin domain (Supplementary Material, Fig. S3), which formed a smaller globular structure with an R_g_ of 15.6 Å and a D_max_ of 55 Å (Fig. 1C and D and Supplementary Material, Fig. S3). Comparison of the pair distribution functions (P(r)) for the retinoschisin monomer and discoidin domain suggested the elongation was a property of the N-terminal Rs1 domain (Fig. 1C). Volumetric modelling confirmed that the long extension within the monomer represented the extended Rs1 domain, forming a ‘wedge’ shape compatible with tight octamerization of the subunits (Fig. 1E).

### Retinoschisin forms a propeller-like planar structure which dimerizes in solution

**Figure 2.**
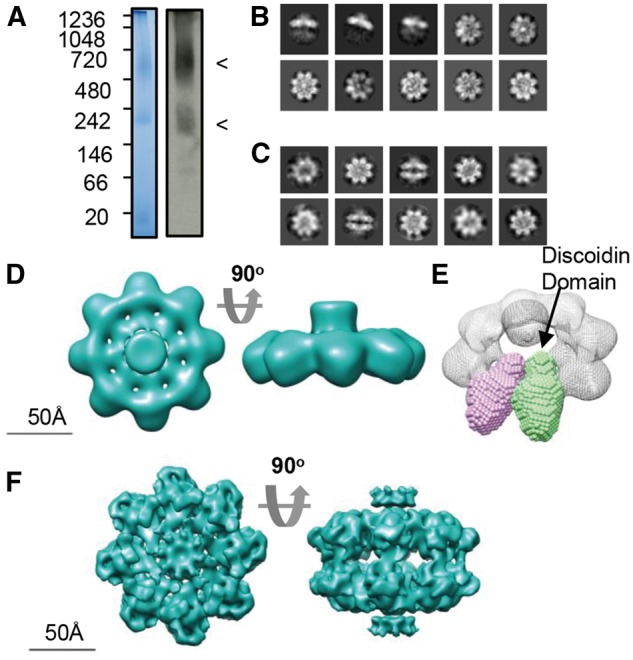
Cryo-TEM analysis of wild-type retinoschisin. (**A**) Blue-native PAGE and native-blot analysis of retinoschisin. Marked are two distinct bands for the octamer and dimer of octamer complexes. (**B**) Reference-free class averages of the octamer and (**C**) dimer of octamers formed in solution. Box size = 24 nm. (**D**) Map of the C8 symmetrical octamer complex at 13.6Å resolution. (**E**) Comparison of the SAXS discoidin domain model to the assembled octamer, the positions of the discoidin domains are shown. (**F**) Map of the retinoschisin dimer of octamers at 9.3Å resolution.

Previously, cryo-EM studies of retinoschisin revealed a hexadecamer formed from two paired octamers (27). Consistent with this, native-PAGE analysis of wild-type retinoschisin showed two species, an octamer and dimer of octamers (Fig. 2A). Using cryo-EM, the octamer structure was determined at 13.6Å resolution and revealed a planar structure (Figs. 2B and D and Supplementary Materials, Figs. S4A and S5) similar to that seen in the hexadecamer, indicating that there is no gross conformational change upon dimerisation. The discoidin domain structure determined by SAXS (Fig. 1D) could be modelled into the octamer in a circular arrangement (Fig. 2E). A higher resolution structure of 9.3Å was achieved (Supplementary Material, Fig. S5) for the dimer of octamers and showed the previously observed double-stack arrangement of octamers (Fig. 2F).

### H207Q destabilizes the retinoschisin monomer and octamer

**Figure 3.**
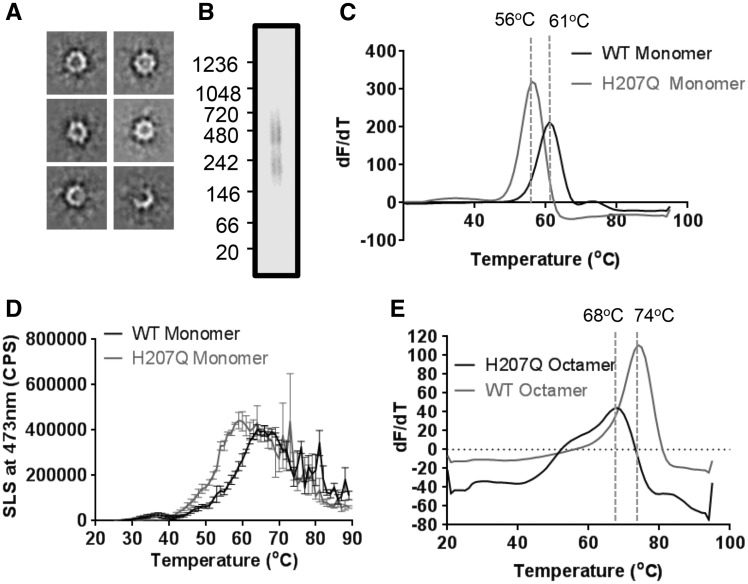
Destabilising effect of the H207Q mutation. (**A**) Reference-free class averages of H207Q octamers under negative-stain conditions. Box size = 35 nm. (**B**) Native-PAGE analysis of H207Q shows two species representing the octamer and dimer of octamers. (**C**) Differential scanning fluorimetry *T_m_* comparison of wild-type and H207Q monomers (*n =* 5). (**D**) Static Light Scattering analysis of wild-type and H207Q monomeric protein during a temperature ramp experiment (*n =* 3) showing *T_agg_* (WT) of 46.2°C and *T_agg_* (H207Q) of 43.5°C. (**E**) Differential scanning fluorimetry *T_m_*comparison of wild-type and H207Q octamers (*n =* 5).

Inspection of the wild-type dimer of octamers complex showed that residue H207 formed a major contact site in the inter-octamer interface suggesting that alteration of octamer dimerization may represent a pathological mechanism. Indeed, the XLRS-associated H207Q mutant retained octameric secretion (22). In order to study the effect of this mutation, the H207Q mutant was expressed and purified. H207Q octamer displayed the characteristic propeller shape using negative-stain EM (Fig. 3A). Furthermore, mutation of this site does not abrogate octamer dimerization. Native-PAGE analysis of H207Q retinoschisin at ∼0.1 mg/ml concentration revealed the formation of the dimer of octamer species (Fig. 3B). However, the mutant assembly was found to be destabilized compared to the wild-type protein. Differential Scanning Fluorimetry (DSF) of monomeric H207Q and wild-type retinoschisin revealed a 5°C reduction in the melting temperature (T_m_) of the mutant protein (Fig. 3C). Intrinsic fluorescence measurements of unfolding confirmed the T_m_ values (Fig. S6). Furthermore, static light scattering (SLS) measurements revealed a greater propensity of the H207Q construct to aggregate in solution conditions (Fig. 3D), with an aggregation temperature of 43.5°C compared to 46.2°C for the wild-type protein and more rapid aggregation reaction. This destabilization was also observed upon octamerisation. Octameric H207Q retinoschisin was found to unfold at a reduced T_m_ (Fig. 3E). Together, these data show that the introduction of the H207Q mutation produces less stable complexes which would serve as less effective structural linkers between retinal cells, leading to pathology.

### Biophysical comparison of wild-type and R141H retinoschisin

**Figure 4.**
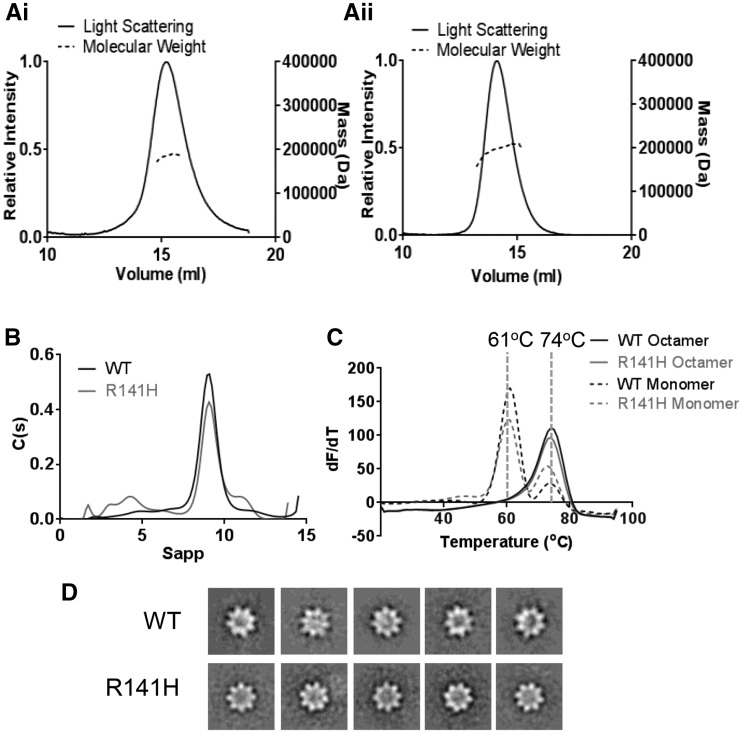
Biophysical comparison of wild-type and R141H retinoschisin. (**A**) SEC-MALS analysis of the wild-type (**i**) and R141H (**ii**) octamer. (**B**) Overlayed sedimentation velocity profiles of wild-type and R141H octamer, both with similar *S_20,w_* (9.38S and 9.79S, respectively), *R_h_* (5.1nm and 5.0 nm, respectively) and *f/f_0_* (1.33 and 1.29 respectively) values. (**C**) Overlayed differential scanning fluorimetry analysis of wild-type and R141H monomers and octamers showing identical *T_m_* for both species (*n =* 5). (**D**) Negatively stained reference-free class averages of wild-type and R141H retinoschisin octamers, box size: 33 nm.

Previously, the disease-causing R141H mutation was found to be secreted as an octamer at similar levels to wild-type retinoschisin (22). However, mutation at this site led to alteration of channel gating kinetics in the L-VGCC binding partner (25). We sought to determine the effect of this mutation on the retinoschisin structure. The R141H retinoschisin octamer revealed a molecular weight of approximately 190 kDa, consistent with the wild-type (Fig. 4A). AUC analysis of wild-type and R141H octamers did not display significant hydrodynamic differences between S_20,w_ (9.38S and 9.79S respectively) and R_h_ (5.1 nm and 5.0 nm respectively) (Fig. 4B). Furthermore, the mutation does not influence the stability of the protein fold or the octameric complex. DSF revealed that both monomeric and octameric wild-type and R141H protein have the same T_m_ (Fig. 4C) which was also observed using intrinsic fluorescence measurements (Fig. S6). Indeed, SLS measurements revealed no increase in aggregation propensity (Fig. S6) and negative stain electron microscopy showed an identical arrangement of the octameric structure (Fig. 4D). Together, these data suggest that unlike H207Q, the R141H mutation does not alter the stability of the complex.

### Cryo-EM structure of the retinoschisin R141H dimer of octamers complex

**Figure 5.**
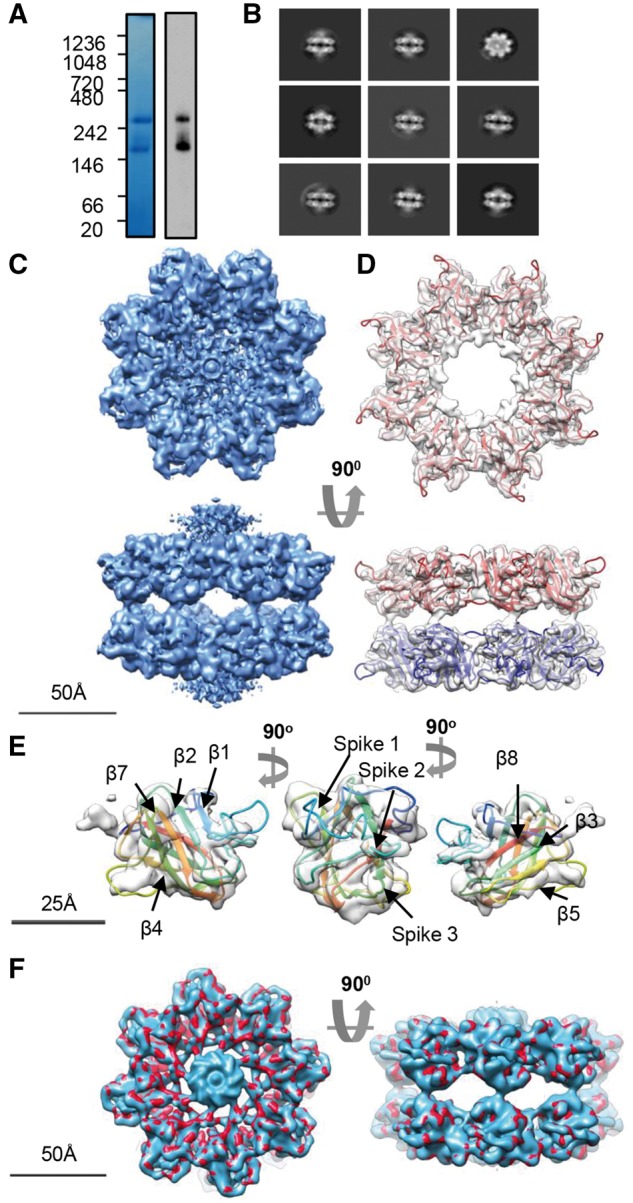
Cryo-EM analysis of the R141H dimer of octamers. (**A**) Blue-native PAGE and native-blot analysis of R141H retinoschisin. (**B**) Reference-free class averages of R141H dimer of octamers. Box size = 33 nm (**C**) Three-dimensional reconstruction at 4.2Å resolution of the R141H dimer of octamers. Shown is the unmasked map. (**D**) Fitting of the R141H discoidin domain model (residues 63-219) shown in red, with associated subunits in the opposing octamer shown in blue. The central density of Rs1 domains has been removed for clarity. (**E**) Fit into single subunit showing the fitting of the β-sheets and positions of the spike regions. (**F**) Comparison between wild-type hexadecamer (*blue, emd_6425*) and R141H retinoschisin (*red*) structures at 5Å resolution.

To investigate further whether there were changes in conformation, the cryo-EM structure of the R141H mutant was investigated. R141H also formed octamer and dimer of octamer species (Fig. 5A), with imaging under cryo-EM conditions confirming a similar double-stack arrangement to the wild-type (Fig. 5B). The number of ‘top’ views of the octamer ring were reduced as there were a greater proportion of side views. Moreover, the increased number of side views allowed for 3D reconstruction of the R141H complex at the higher resolution of 4.2Å (Fig. 5C and Supplementary Material, Fig. S7). This revealed a comparable domain arrangement to the wild-type protein. A homology model of the R141H discoidin domain was constructed and fitted to the map using DockEM (Fig. 5D). The highest correlation fit (9.77 sigma above the mean cross-correlation value) was then refined by flexible fitting using the FlexEM programme (28). This yielded a final fit with a correlation of 0.89 at 4.2Å resolution, determined by UCSF Chimera. As previously observed (27), the central density represented the flexible N-terminal Rs1 domains which were masked out in the refinement procedure (Fig. 5D and Supplementary Material, Fig. S7). In this model, the spike regions project towards the propeller tips (Figs. 5E). Comparison of the R141H hexadecamer structure to the wild-type retinoschisin map (27) filtered at 5Å with DockEM, revealed a highly similar structure (Fig. 5F).

### Identification of three distinct classes of XLRS mutations

The identification of complex interfaces using PDBePISA analysis (Supplementary Material, Fig. S8) allowed for interpretation of the effect of mutations upon the assembled retinoschisin molecule. Non-cysteine XLRS-associated mutations which caused conservative changes or were previously predicted to have negligible effects on monomer folding (29,30) were mapped onto the structure (Supplementary Material, Fig. S9). One identified class of mutations clustered at the intra-octamer interface (Supplementary Material, Fig. S9A). Indeed, E72, N104 and T185 were implicated in direct contact between the domains (Supplementary Material, Fig. S9), with both E72K and T185K mutations retained intracellularly (21,22). Another class of conservative mutations was located at the inter-octamer interface. Two contact sites were previously suggested (27) and here an additional contact site is identified. This third site between the octamer rings is formed by the loop between strands β4 and β5 (residues 178–182). All three contact sites have conservative mutations which lead to XLRS, these include residues in strand β4 (D145 and E146), the proximal loop region (G178, and N179) as well as strand β7 (H207 and R209) (Supplementary Material, Fig. S9B).

**Figure 6.**
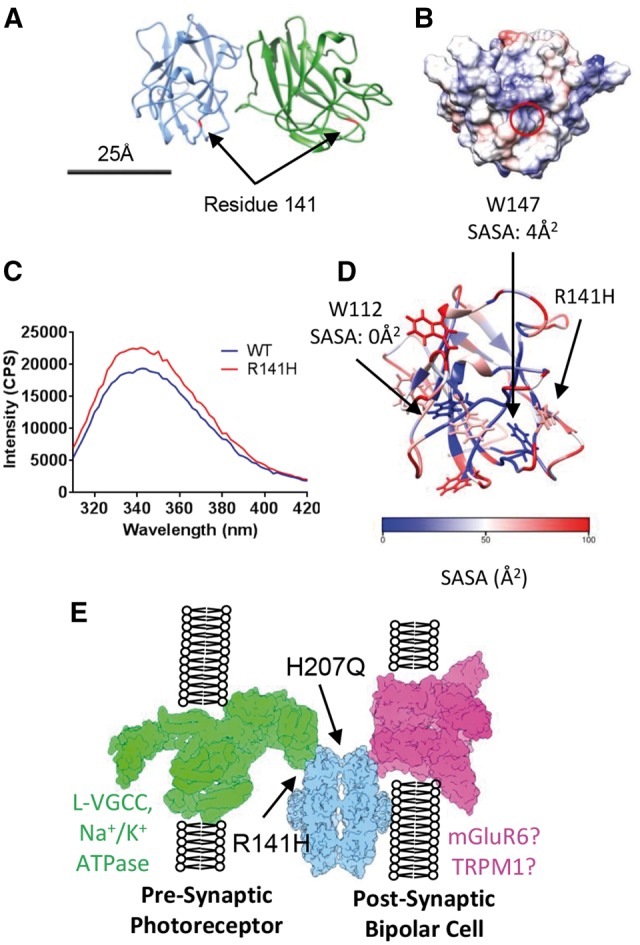
Mapping XLRS-causative conservative mutations onto the quasi-atomic model. (**A**) The position of residue R141 within the octamer is highlighted in red. (**B**) Electrostatic surface potential of the face of the discoidin domain containing residue R141 (circled) with overall positive charge. Scale bar for (A) and (B) = 25 nm. (**C**) Intrinsic fluorescence comparison of wild-type and R141H retinoschisin monomer. The R141H mutant has reduced fluorescence quenching suggesting increased solvent exposure of tryptophans. Shown is the emission spectrum after excitation at 295nm (*n =* 3). (**D**) Solvent accessible surface area (SASA) analysis of the tryptophans in the retinoschisin discoidin domain. W147 and W112 are buried and close to the R141H mutation site. (**E**) The paired octamer model for retinoschisin structural support between photoreceptor and bipolar cell synaptic membranes, with the sites affected by R141H and H207Q mutations labelled.

Mapping of the R141H mutation indicated that it was located in a spike region distinct from intra- and inter-octamer interfaces (Fig. 6A) with an overall positive charge (Fig. 6B). Comparison of the intrinsic fluorescence of wild-type and R141H mutant retinoschisin showed increased fluorescence in the mutant despite the lack of gross conformational change (Fig. 6C), suggesting a small change in the spike regions. Indeed, analysis of the homology model revealed two buried tryptophan residues in the model (W112 and W147) found in spikes 2 and 3 respectively, within 1 nm of the mutation site (Fig. 6D). This suggests the R141H mutation leads to a subtle conformational change within this region, however lowered local resolution in the electron density maps precluded observation of this alteration. Additionally, R141H has discrete bands visualized by native-PAGE (Fig. 5A) whereas the wild-type and H207Q mutant run as diffuse broad bands (Figs. 2A and 3B) suggesting that the R141H mutant changes the charge or conformation of the complex leading to altered mobility in native-PAGE. Together these subtle alterations may disrupt a binding interface within the propeller tips (Fig. 6E).

## Discussion

Despite the central role retinoschisin plays in the maintenance of retinal architecture, the mechanism by which it maintains the integrity of photoreceptor-bipolar cell synapses remains elusive. Therefore, investigations into the impact of XLRS-associated mutations upon the structure are critical to understand the molecular function of retinoschisin.

SAXS analysis of the retinoschisin monomer displayed a highly elongated structure; the extended conformation could be attributed to the N-terminal Rs1 domain with the discoidin domain having a globular structure (Fig. 1E). This generates a ‘wedge’ shape which would allow for efficient assembly of the retinoschisin subunits into a ring structure. Indeed, the solution of the retinoschisin octamer structure revealed a ‘ring’ of disulfide-stabilized discoidin domains which form a planar structure (Figs. 2D and E), forming a flat interface for dimerization of the octamer rings (Fig. 2F). As previously described (27) and as we also observe, the central Rs1 domains formed a diffuse unstructured region in the retinoschisin hexadecamer, together with the extended volume for this region in the retinoschisin monomer suggests that the Rs1 domain intrinsically lacks a defined structure. The lower resolution or heavy-metal staining artefacts could explain the absence of this density in the negative stain structure determined previously (26).

The observation of mutation sites within the inter-octamer interface lead to the hypothesis that the hexadecamer complex may be required for retinoschisin function in the retina. In particular, H207 was found to prominently feature in this interface with conservative point mutations (such as H207Q) associated with the development of XLRS (5), despite being secreted as an octameric complex (22). In order to determine the effect of this mutation, the H207Q mutant retinoschisin complex was isolated. The mutant complex retained the same shape and arrangement of domains within the octamer as the wild-type (Fig. 3). Native-PAGE analysis showed that the mutant protein formed dimers of octamers. However, further investigation into the unfolding and aggregation behaviour of the protein revealed that the introduction of the H207Q mutation led to a destabilization of the monomeric and octameric structures (Fig. 3C–E). Hence producing a less stable structural support must lead to the development of XLRS pathology. Together, these data support the paired octamer model for retinoschisin-mediated structural support. Retinoschisin has previously been shown to form structures which span adjacent photoreceptor membranes (31) and cluster proteins essential in neurotransmission at the photoreceptor-bipolar cell synapse (32). The formation of a retinoschisin dimer of octamers complex (Fig. 6E) would allow for co-localization of synaptic machinery on opposing membranes whilst providing structural support to the synapse. Loss or destabilization of the complex would lead to loss of structural integrity and pathology. Employing PDBePISA analysis, interfaces and mutations were identified both within the octamer between the discoidin domains and between the octamer rings (Supplementary Materials, Figs. S8 and S9). Additionally, PDBePISA analysis identified another interface between the octamers, the loop between β4 and β5 (residues 178-182). This site has two conservative mutations associated with the development of XLRS (G178D and N179D) that we predict to destabilize the inter-octamer interface.

The paired octamers model for retinoschisin assembly also relies upon interfaces contacting with opposing membranes. Previously, it has been shown that the R141S mutation leads to a loss of galactose binding (33) with another mutation which retained octameric secretion, R141G, altering the channel gating kinetics of a known binding partner L-VGCC (25). Determination of the mutant R141H complex structure to 4.2Å resolution revealed a highly similar structure to the wild-type protein, consistent with biophysical comparisons. Despite the lack of gross conformational change, comparison of wild-type and R141H retinoschisin monomers revealed an altered intrinsic fluorescence, with a reduced fluorescence quenching in the mutant suggesting increased solvent exposure of tryptophans (Figs. 6B and C). Closer inspection of the discoidin domain model revealed only two buried tryptophans within the structure (W112 and W147) both within 1 nm of R141. W147 is immediately proximal to the mutation site on spike 3, whereas W112 is found on spike 2. Mutation of R141 appears to increase solvent accessibility of one or both of these tryptophans. The disulfide-linkage between spikes 3 and 2 (10) could act to propagate a conformational change in spike 3 to spike 2, generating a wider subtle reorganization of the spikes at the propeller tips. This may disrupt the interaction site in this region, altering the interaction with L-VGCC and other binding partners. Indeed, an alteration in the surface charge induced by this mutation could alter interactions with calcium ions, which have been demonstrated to facilitate retinoschisin association with membranes (34). Therefore, future studies should concentrate on structurally characterizing these interactions in order to identify the nature of the effect of R141H on interactions with L-VGCC or other binding partners. In summary, we show effects of two classes of secreted XLRS mutations on the structure and stability of retinoschisin to provide further insights into their pathology.

## Materials and Methods

### Protein Expression and Purification

Wild-type, R141H and H207Q C-terminally His-tagged human retinoschisin was expressed in HEK293-EBNA cells stably transfected with the pCEP-Pu/AC7 vector. Cells were cultured as described previously (35). Wild-type, C-terminally His-tagged Discoidin domain was expressed in *Pichia pastoris* X-33 (Life Technologies) stably transformed with pPICZA-α vector (Life Technologies). Transformed cells were grown in 10ml of BMGY medium and transferred to BMMY medium containing 0.5% methanol for induction. Cells were grown at 30°C under rotation for 72 h. Expressed protein was purified using Ni-affinity chromatography followed by size exclusion chromatography using a Superdex75 10/300 (GE Healthcare) pre-equilibrated with 10 mM Tris pH7.4, 150 mM NaCl at 0.5 ml/min.

### MALS Analysis

Purified samples of 0.5 ml volume were loaded onto a Superdex75 10/300GL column (GE Healthcare) at 0.75 ml/min in 10 mM Tris pH 7.4, 150 mM NaCl and passed through a Wyatt DAWN Heleos II EOS 18-angle laser photometer coupled to a Wyatt Optilab rEX refractive index detector. Resulting hydrodynamic radii and molecular mass measurements were analysed using Astra 6.

### Sedimentation Velocity AUC

Wild-type and mutant retinoschisin (∼0.1 mg/ml) in 10 mM Tris pH 7.4, 150 mM NaCl were centrifuged simultaneously using a XL-A centrifuge (Beckman) at 45,000 rpm in an An60Ti-4 Hole rotor at 20°C with the sedimenting boundary monitored at 230 nm every 3 min for 250 scans. Data were analysed using continuous model-based distribution of Lamm equation solutions (C(s)) using the Sedfit software suite (36).

### SAXS Data Collection and Analysis

Data were collected at beamline BM29, European Synchrotron Radiation Facility, Grenoble, France. Purified discoidin domain (50 µl at 1 mg/ml) was passed through a Superdex200 Increase 3.2/300 at 0.1 ml/min in 10 mM Tris pH 7.4, 150 mM NaCl, with protein elution monitored using A_280_. Data were collected using the Pilatus 1M detector at a 2.8m distance, using a q-range between 0.01 and 5 nm^−1^. Data were reduced using in-house software and buffer subtraction and analysis was performed using the ScÅtter software package (http://www.bioisis.net/; date last accessed October 17, 2016). Volumetric modelling was carried out as described in (37).

### Intrinsic Fluorescence Spectroscopy

Intrinsic fluorescence measurements were carried out on 12 µg of purified wild-type and R141H retinoschisin monomers at 0.1 mg/ml in 120 µl of 10 mM Tris pH7.4, 150 mM NaCl. Three-dimensional spectra (excitation between 240 nm-305 nm with emission between 310 nm and 420 nm recorded) were measured using a Horiba Fluoromax flourimeter.

### Negative Stain TEM and Image Processing

3 µl of 20 µg/ml octamer was adsorbed onto carbon coated grids (Agar Scientific) glow discharged at 25mA for 25 s. The sample was stained with Uranyl Acetate and imaged using an FEI Tecnai12 Biotwin electron microscope operating at 120kV as described in (37). Micrographs were analysed using the EMAN2.0 (38). Particles were picked using a box size of 33, 33 and 35 nm giving final data sets of 6665, 10,108 and 1985 particles for wild-type, R141H and H207Q respectively, which was subject to iterative alignment and classification.

### Cryo-EM Data Collection and Image Processing

3 µl of 0.1 mg/ml of octamer was vitirified on a C-Flat R2/2 copper grid (EMS). The wild-type octamer was imaged in an FEI Tecnai F30 coupled with a Gatan Quantum K2 Summit direct electron counting camera. Images were recorded at 59 000x nominal magnification, giving a pixel size of 0.8Å/pixel at the specimen level. 120 movie mode images were recorded in which the sample was irradiated with a total dose of 25e^-^/Å^2^ for 25 frames at 0.1 s/frame over a defocus range of –1 µm to –4 µm. R141H retinoschisin octamer images were recorded at the electron Bio-Imaging Centre (eBIC) using an FEI Titan Krios equipped with a Gatan Quantum K2 Summit detector. Images were recorded with EPU (FEI) at 105 000x nominal magnification in EFTEM, giving a pixel size of 1.29 Å/pixel at the specimen level. Seven-second exposures were fractionated into 500ms frames using a dose rate of 5 e^-^/pixel/s for a total dose of 40e^-^/Å^2^ and a defocus range of -1 to -4.5 µm for a total of 1200 movie mode images. Movie exposures were stacked and particle drift corrected using Motioncorr (39). Wild-type images were 2x2 Fourier binned to 1.6Å/pixel. Particles were selected using EMAN2.0 generating particle sets of 14,614 and 13,343 particles for wild-type and R141H respectively. Particles were class averaged using RELION1.4 (40). Particle heterogeneity was minimized by class averaging (discarding 11,829 and 6287 particles respectively). Wild-type particles were three-dimensionally classified into 2 classes using C8 symmetry for wild-type particle set, generating classes for the octamer (1320 particles) and dimer of octamers (1465 particles). No single stack octamers were observed following class averaging of the R141H particle set. Therefore, D8 symmetry was used for three-dimensional classification into two classes. Variation was only observed in the disordered central region, which was subsequently masked out during refinement, therefore a single D8 symmetrical three-dimensional class (containing 7056 particles) was used for further refinement. At an intermediate stage prior to this refinement, particles were re-centred using the recenter.py python script (41) to generate a new RELION star file, before further rounds of refinement were performed. The R141H map was refined further using a soft Gaussian mask excluding the central Rs1 domain region. The final resolution was determined using the Fourier Shell Correlation (FSC) at the 0.5 and 0.143 criterion. Local resolution in resulting maps was further analysed using ResMap-H2 (42), with local resolution distribution visualized in UCSF Chimera (43).

### Homology Modelling

A homology model of the retinoschisin discoidin domain (residues 63-219) was constructed using the Phyre2 Protein Fold Recognition Server (44). Disulfide bonds were inserted and the energy minimized using UCSF Chimera.

### Domain Fitting and Refinement

Domain fitting was carried out using DockEM (45). The discoidin domain homology model was Fourier filtered to 4.5Å, and a filtered version of the map (at 4.5Å) was globally searched at 4°' angular increments. A local mask defining the footprint of the discoidin domain was calculated by convoluting a 10Å sphere with the domain density filtered to 10Å. A threshold defining a mask boundary that encompassed the 4.5Å domain density closely was chosen in UCSF Chimera. A top fit was obtained, with a correlation coefficient 9.77 standard deviations above the mean value for all observed fits. The next highest non-equivalent fit had a value of 3.1 standard deviations above the mean. The top scoring fit was flexibly refined to the structure using FlexEM flexible fitting (28) with four iterations of molecular dynamic optimisation. The fit was then refined using the symmetrical fitting option in UCSF Chimera, giving a final correlation of 0.89 at 4.2Å resolution. Handedness was confirmed by fitting the homology model to the opposite hand of the map using DockEM. The highest correlation fit with the mirrored structure revealed a lower statistical significance of 4.1 standard deviations above the mean, with a final flexibly fitted correlation value reduced by 0.2 relative to the fit to the opposing hand. Interfaces were identified using the PDBePISA webserver (46) (http://www.ebi.ac.uk/pdbe/pisa/; date last accessed October 17, 2016). A new version of DockEM is available from www.ccpem.ac.uk; date last accessed October 17, 2016.

### DockEM Map Comparison

Both wild-type (emd_6425) and R141H retinoschisin hexadecamer maps were compared using DockEM. The absolute magnification of the R141H map was optimized by the local fitting of the discoidin domain homology model (derived from atomic coordinates) to the maps, using DockEM with a scale search option. For this, the map and model were Fourier filtered to 5Å. The search was carried out rotating the domain up to 20° in 4° increments, starting from the previously fitted position. The domain was re-scaled by a factor of 0.9 to 1.1 in 1% increments. The best correlation of the search returned a position and orientation of the domain close to the starting position. For the R141H map the correlation search indicated that a 0.95 scale factor needed to be applied to give the best match between the map calculated from the atomic structure of the domain, and the EM map, indicating an updated sampling of 1.35 Å/pixel. The R141H and wild-type maps were compared in DockEM using a similar procedure, giving a relative scale of 0.96, implying the sampling of the wild-type map to be 0.99 Å/pixel. The optimum relative magnification obtained allowed for accurate comparison of the maps. The updated samplings of the maps computed were verified by visual inspection of the maps and fits in UCSF Chimera, and the quality of fit was improved.

### Differential Scanning Fluorimetry

Measurements were carried out using a CFX96 Real Time PCR Detection system (Bio Rad) in a total volume of 10 µl consisting of 9 µl of wild-type or R141H octamer or monomer at 0.1 mg/ml concentration with 1 µl of Sypro Orange (Life Technologies) at a final concentration of 10x as previously described (47).

### OPTIM Stability Analysis

Monitoring of protein folding using barycentric mean fluorescence (BCM) measurements and aggregation (using static light scattering at 473 nm wavelength) was carried out using the OPTIM1000 (Avacta Analytical). 9 µl of 0.1 mg/ml protein in 10 mM Tris, 150 mM NaCl at pH7.4 was incubated at 20°C in an OPTIM microcuvette. Temperature was increased at 1°C intervals to 90°C.

## Supplementary Material

Supplementary Material is available at *HMG* online.

## Supplementary Material

Supplementary Data

## References

[1] Hoon M. Okawa H. Della Santina L. Wong R.O. (2014) Functional architecture of the retina: development and disease. Prog. Retin. Eye Res., 42, 44–84. 24984227 10.1016/j.preteyeres.2014.06.003 PMC4134977

[2] Sikkink S.K. Biswas S. Parry N.R. Stanga P.E. Trump D. (2007) X-linked retinoschisis: an update. J. Med. Genet., 44, 225–232. 17172462 10.1136/jmg.2006.047340 PMC2598044

[3] Stenson P.D. Mort M. Ball E.V. Shaw K. Phillips A. Cooper D.N. (2014) The Human Gene Mutation Database: building a comprehensive mutation repository for clinical and molecular genetics, diagnostic testing and personalized genomic medicine. Hum. Genet., 133, 1–9. 24077912 10.1007/s00439-013-1358-4 PMC3898141

[4] Pimenides D. George N.D. Yates J.R. Bradshaw K. Roberts S.A. Moore A.T. Trump D. (2005) X-linked retinoschisis: clinical phenotype and RS1 genotype in 86 UK patients. J. Med. Genet., 42, e35. 15937075 10.1136/jmg.2004.029769 PMC1736077

[5] den Dunnen J.T. Kraayenbrink T. van Schooneveld M. van de Vosse E. de Jong P.T.V.M. ten Brink J.B. Schuurman E. Tijmes N. van Ommen G.J.B. Bergen A.A.B., (1998) Functional implications of the spectrum of mutations found in 234 cases with X-linked juvenile retinoschisis (XLRS). Hum. Mol. Genet., 7, 1185–1192. 9618178 10.1093/hmg/7.7.1185

[6] Sauer C.G. Gehrig A. Warneke-Wittstock R. Marquardt A. Ewing C.C. Gibson A. Lorenz B. Jurklies B. Weber B.H. (1997) Positional cloning of the gene associated with X-linked juvenile retinoschisis. Nat. Genet., 17, 164–170. 9326935 10.1038/ng1097-164

[7] Reid S.N. Akhmedov N.B. Piriev N.I. Kozak C.A. Danciger M. Farber D.B. (1999) The mouse X-linked juvenile retinoschisis cDNA: expression in photoreceptors. Gene, 227, 257–266. 10023077 10.1016/s0378-1119(98)00578-2

[8] Grayson C. Reid S.N. Ellis J.A. Rutherford A. Sowden J.C. Yates J.R. Farber D.B. Trump D. (2000) Retinoschisin, the X-linked retinoschisis protein, is a secreted photoreceptor protein, and is expressed and released by Weri-Rb1 cells. Hum. Mol. Genet., 9, 1873–1879. 10915776 10.1093/hmg/9.12.1873

[9] Kiedzierska A. Smietana K. Czepczynska H. Otlewski J. (2007) Structural similarities and functional diversity of eukaryotic discoidin-like domains. Biochim. Biophys. Acta, 1774, 1069–1078. 17702679 10.1016/j.bbapap.2007.07.007

[10] Wu W.W. Wong J.P. Kast J. Molday R.S. (2005) RS1, a discoidin domain-containing retinal cell adhesion protein associated with X-linked retinoschisis, exists as a novel disulfide-linked octamer. J. Biol. Chem., 280, 10721–10730. 15644328 10.1074/jbc.M413117200

[11] Reid S.N. Yamashita C. Farber D.B. (2003) Retinoschisin, a photoreceptor-secreted protein, and its interaction with bipolar and muller cells. J. Neurosci., 23, 6030–6040. 12853421 10.1523/JNEUROSCI.23-14-06030.2003 PMC6740352

[12] Takada Y. Fariss R.N. Tanikawa A. Zeng Y. Carper D. Bush R. Sieving P.A. (2004) A retinal neuronal developmental wave of retinoschisin expression begins in ganglion cells during layer formation. Invest. Ophthalmol. Vis. Sci., 45, 3302–3312. 15326155 10.1167/iovs.04-0156

[13] Weber B.H. Schrewe H. Molday L.L. Gehrig A. White K.L. Seeliger M.W. Jaissle G.B. Friedburg C. Tamm E. Molday R.S. (2002) Inactivation of the murine X-linked juvenile retinoschisis gene, Rs1h, suggests a role of retinoschisin in retinal cell layer organization and synaptic structure. Proc. Natl. Acad. Sci. U S A, 99, 6222–6227. 11983912 10.1073/pnas.092528599 PMC122930

[14] Zeng Y. Takada Y. Kjellstrom S. Hiriyanna K. Tanikawa A. Wawrousek E. Smaoui N. Caruso R. Bush R.A. Sieving P.A. (2004) RS-1 Gene Delivery to an Adult Rs1h Knockout Mouse Model Restores ERG b-Wave with Reversal of the Electronegative Waveform of X-Linked Retinoschisis. Invest. Ophthalmol. Vis. Sci., 45, 3279–3285. 15326152 10.1167/iovs.04-0576

[15] Min S.H. Molday L.L. Seeliger M.W. Dinculescu A. Timmers A.M. Janssen A. Tonagel F. Tanimoto N. Weber B.H. Molday R.S., (2005) Prolonged recovery of retinal structure/function after gene therapy in an Rs1h-deficient mouse model of x-linked juvenile retinoschisis. Mol. Ther., 12, 644–651. 16027044 10.1016/j.ymthe.2005.06.002

[16] Kjellstrom S. Bush R.A. Zeng Y. Takada Y. Sieving P.A. (2007) Retinoschisin gene therapy and natural history in the Rs1h-KO mouse: long-term rescue from retinal degeneration. Invest. Ophthalmol. Vis. Sci., 48, 3837–3845. 17652759 10.1167/iovs.07-0203

[17] Janssen A. Min S.H. Molday L.L. Tanimoto N. Seeliger M.W. Hauswirth W.W. Molday R.S. Weber B.H. (2008) Effect of late-stage therapy on disease progression in AAV-mediated rescue of photoreceptor cells in the retinoschisin-deficient mouse. Mol. Ther., 16, 1010–1017. 18388913 10.1038/mt.2008.57

[18] Park T.K. Wu Z. Kjellstrom S. Zeng Y. Bush R.A. Sieving P.A. Colosi P. (2009) Intravitreal delivery of AAV8 retinoschisin results in cell type-specific gene expression and retinal rescue in the Rs1-KO mouse. Gene Ther., 16, 916–926. 19458650 10.1038/gt.2009.61 PMC2774250

[19] Wang T. Waters C.T. Rothman A.M. Jakins T.J. Romisch K. Trump D. (2002) Intracellular retention of mutant retinoschisin is the pathological mechanism underlying X-linked retinoschisis. Hum. Mol. Genet., 11, 3097–3105. 12417531 10.1093/hmg/11.24.3097

[20] Wu W.W. Molday R.S. (2003) Defective discoidin domain structure, subunit assembly, and endoplasmic reticulum processing of retinoschisin are primary mechanisms responsible for X-linked retinoschisis. J. Biol. Chem., 278, 28139–28146. 12746437 10.1074/jbc.M302464200

[21] Dyka F.M. Molday R.S. (2007) Coexpression and interaction of wild-type and missense RS1 mutants associated with X-linked retinoschisis: its relevance to gene therapy. Invest. Ophthalmol. Vis. Sci., 48, 2491–2497. 17525175 10.1167/iovs.06-1465

[22] Wang T. Zhou A. Waters C.T. O'Connor E. Read R.J. Trump D. (2006) Molecular pathology of X linked retinoschisis: mutations interfere with retinoschisin secretion and oligomerisation. Br. J. Ophthalmol., 90, 81–86. 16361673 10.1136/bjo.2005.078048 PMC1856892

[23] Gleghorn L.J. Trump D. Bulleid N.J. (2010) Wild-type and missense mutants of retinoschisin co-assemble resulting in either intracellular retention or incorrect assembly of the functionally active octamer. Biochem. J., 425, 275–283. 10.1042/BJ20091179 19849666

[24] Molday L.L. Wu W.W. Molday R.S. (2007) Retinoschisin (RS1), the protein encoded by the X-linked retinoschisis gene, is anchored to the surface of retinal photoreceptor and bipolar cells through its interactions with a Na/K ATPase-SARM1 complex. J. Biol. Chem., 282, 32792–32801. 17804407 10.1074/jbc.M706321200

[25] Shi L. Jian K. Ko M.L. Trump D. Ko G.Y. (2009) Retinoschisin, a new binding partner for L-type voltage-gated calcium channels in the retina. J. Biol. Chem., 284, 3966–3975. 19074145 10.1074/jbc.M806333200 PMC2635055

[26] Bush M. Setiaputra D. Yip C.K. Molday R.S. (2016) Cog-wheel octameric structure of RS1, the discoidin domain containing retinal protein associated with X-linked retinoschisis. PLoS One, 11, e0147653. 26812435 10.1371/journal.pone.0147653 PMC4728063

[27] Tolun G. Vijayasarathy C. Huang R. Zeng Y. Li Y. Steven A.C. Sieving P.A. Heymann J.B. (2016) Paired octamer rings of retinoschisin suggest a junctional model for cell-cell adhesion in the retina. Proc. Natl. Acad. Sci. U S A, 113, 5287–5292. 27114531 10.1073/pnas.1519048113 PMC4868477

[28] Topf M. Lasker K. Webb B. Wolfson H. Chiu W. Sali A. (2008) Protein structure fitting and refinement guided by cryo-EM density. Structure, 16, 295–307. 18275820 10.1016/j.str.2007.11.016 PMC2409374

[29] Sergeev Y.V. Caruso R.C. Meltzer M.R. Smaoui N. MacDonald I.M. Sieving P.A. (2010) Molecular modeling of retinoschisin with functional analysis of pathogenic mutations from human X-linked retinoschisis. Hum. Mol. Genet., 19, 1302–1313. 20061330 10.1093/hmg/ddq006 PMC2838538

[30] Sergeev Y.V. Vitale S. Sieving P.A. Vincent A. Robson A.G. Moore A.T. Webster A.R. Holder G.E. (2013) Molecular modeling indicates distinct classes of missense variants with mild and severe XLRS phenotypes. Hum. Mol. Genet., 22, 4756–4767. 23847049 10.1093/hmg/ddt329 PMC3820135

[31] Vijayasarathy C. Takada Y. Zeng Y. Bush R.A. Sieving P.A. (2007) Retinoschisin is a peripheral membrane protein with affinity for anionic phospholipids and affected by divalent cations. Invest. Ophthalmol. Vis. Sci., 48, 991–1000. 17325137 10.1167/iovs.06-0915

[32] Ou J. Vijayasarathy C. Ziccardi L. Chen S. Zeng Y. Marangoni D. Pope J.G. Bush R.A. Wu Z. Li W., (2015) Synaptic pathology and therapeutic repair in adult retinoschisis mouse by AAV-RS1 transfer. J. Clin. Invest., 125, 2891–2903. 26098217 10.1172/JCI81380 PMC4563692

[33] Dyka F.M. Wu W.W. Pfeifer T.A. Molday L.L. Grigliatti T.A. Molday R.S. (2008) Characterization and purification of the discoidin domain-containing protein retinoschisin and its interaction with galactose. Biochemistry, 47, 9098–9106. 18690710 10.1021/bi800938g PMC2651826

[34] Kotova S. Vijayasarathy C. Dimitriadis E.K. Ikonomou L. Jaffe H. Sieving P.A. (2010) Retinoschisin (RS1) interacts with negatively charged lipid bilayers in the presence of Ca2+: an atomic force microscopy study. Biochemistry, 49, 7023–7032. 20677810 10.1021/bi1007029 PMC2929131

[35] Berry R. Jowitt T.A. Ferrand J. Roessle M. Grossmann J.G. Canty-Laird E.G. Kammerer R.A. Kadler K.E. Baldock C. (2009) Role of dimerization and substrate exclusion in the regulation of bone morphogenetic protein-1 and mammalian tolloid. Proc. Natl. Acad. Sci. U S A, 106, 8561–8566. 19429706 10.1073/pnas.0812178106 PMC2689009

[36] Schuck P. (2000) Size-distribution analysis of macromolecules by sedimentation velocity ultracentrifugation and lamm equation modeling. Biophys. J., 78, 1606–1619. 10692345 10.1016/S0006-3495(00)76713-0 PMC1300758

[37] Troilo H. Zuk A.V. Tunnicliffe R.B. Wohl A.P. Berry R. Collins R.F. Jowitt T.A. Sengle G. Baldock C. (2014) Nanoscale structure of the BMP antagonist chordin supports cooperative BMP binding. Proc. Natl. Acad. Sci. U S A, 111, 13063–13068. 25157165 10.1073/pnas.1404166111 PMC4246984

[38] Tang G. Peng L. Baldwin P.R. Mann D.S. Jiang W. Rees I. Ludtke S.J. (2007) EMAN2: an extensible image processing suite for electron microscopy. J. Struct. Biol., 157, 38–46. 16859925 10.1016/j.jsb.2006.05.009

[39] Li X. Mooney P. Zheng S. Booth C.R. Braunfeld M.B. Gubbens S. Agard D.A. Cheng Y. (2013) Electron counting and beam-induced motion correction enable near-atomic-resolution single-particle cryo-EM. Nat. Methods, 10, 584–590. 23644547 10.1038/nmeth.2472 PMC3684049

[40] Scheres S.H. (2012) RELION: implementation of a Bayesian approach to cryo-EM structure determination. J. Struct. Biol., 180, 519–530. 23000701 10.1016/j.jsb.2012.09.006 PMC3690530

[41] Rawson S. Iadanza M.G. Ranson N.A. Muench S.P. (2016) Methods to account for movement and flexibility in cryo-EM data processing. Methods, 100, 35–41. 27016144 10.1016/j.ymeth.2016.03.011 PMC4854228

[42] Kucukelbir A. Sigworth F.J. Tagare H.D. (2014) Quantifying the local resolution of cryo-EM density maps. Nat. Methods, 11, 63–65. 24213166 10.1038/nmeth.2727 PMC3903095

[43] Pettersen E.F. Goddard T.D. Huang C.C. Couch G.S. Greenblatt D.M. Meng E.C. Ferrin T.E. (2004) UCSF Chimera–a visualization system for exploratory research and analysis. J. Comput. Chem., 25, 1605–1612. 15264254 10.1002/jcc.20084

[44] Kelley L.A. Mezulis S. Yates C.M. Wass M.N. Sternberg M.J. (2015) The Phyre2 web portal for protein modeling, prediction and analysis. Nat. Protoc., 10, 845–858. 25950237 10.1038/nprot.2015.053 PMC5298202

[45] Roseman A.M. (2000) Docking structures of domains into maps from cryo-electron microscopy using local correlation. Acta Crystallogr. D., 56, 1332–1340. 10998630 10.1107/s0907444900010908

[46] Krissinel E. Henrick K. (2007) Inference of macromolecular assemblies from crystalline state. J. Mol. Biol., 372, 774–797. 17681537 10.1016/j.jmb.2007.05.022

[47] Chari A. Haselbach D. Kirves J.M. Ohmer J. Paknia E. Fischer N. Ganichkin O. Moller V. Frye J.J. Petzold G., (2015) ProteoPlex: stability optimization of macromolecular complexes by sparse-matrix screening of chemical space. Nat. Methods, 12, 859–865. 26237227 10.1038/nmeth.3493 PMC5136620

