# Supplementary material for: Structural analysis of X-linked retinoschisis mutations reveals distinct classes which differentially effect retinoschisin function
Source: Hum Mol Genet. 2016 Oct 23;25(24):5311–20. doi: 10.1093/hmg/ddw345 (PMC5418834; doi:10.1093/hmg/ddw345)
Supplement: Supplementary Data [file ddw345_Supp.pdf]

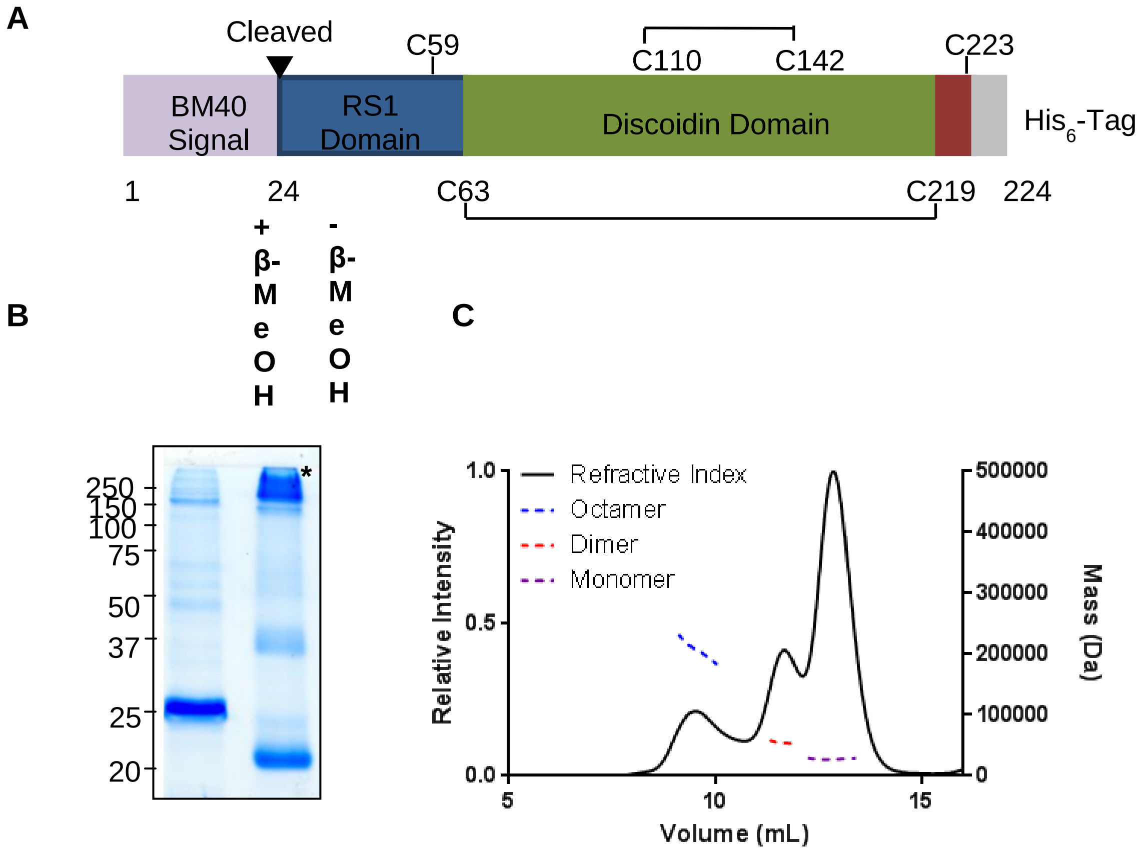

Supplementary Figure 1 – Purification of wild-type retinoschisin. (**A**) Schematic diagram of the retinoschisin construct used for these studies, with residues required for disulphide formation marked. (**B**) SDS-PAGE gel of retinoschisin after Ni-NTA affinity purification, disulphide-dependent octamer formation indicated by *. (**C**) SEC-MALS purification of retinoschisin, showing separation of Octamer, Dimer and Monomer species with molecular weights of ~200, 54 and 27kDa respectively.

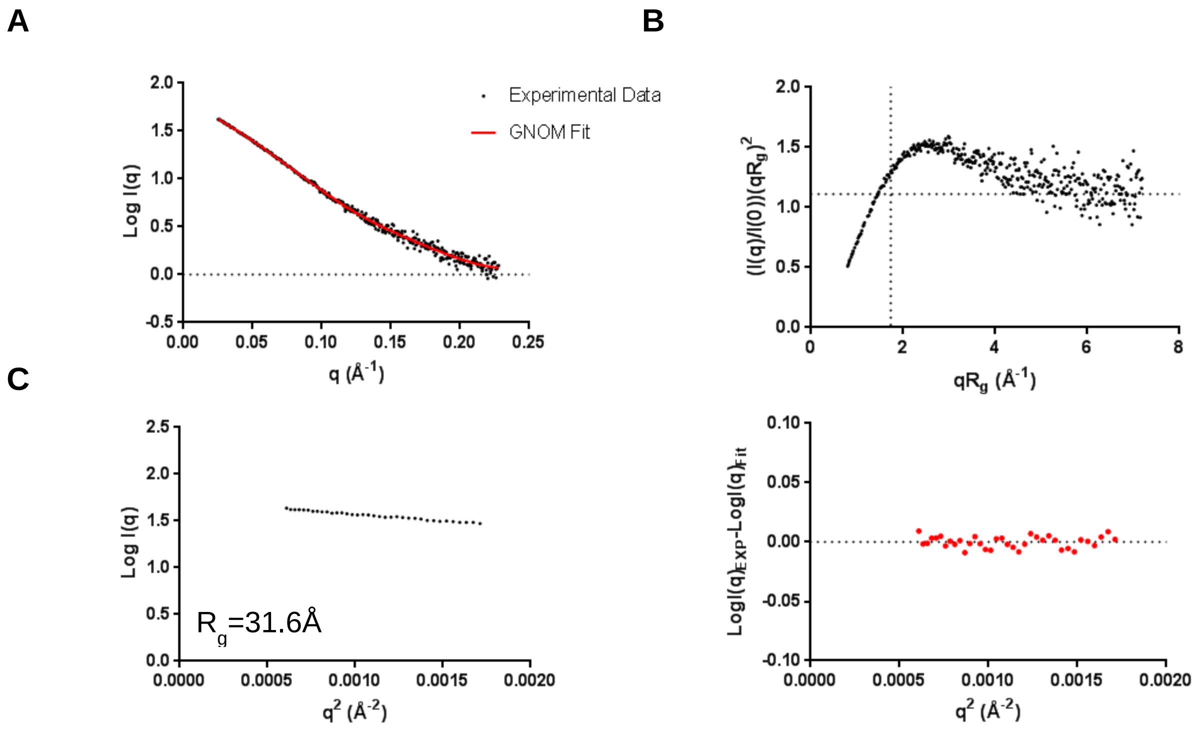

Supplementary Figure 2 – SAXS analysis of wild-type retinoschisin monomer. (**A**) The rotationally averaged scatter profile of purified retinoschisin monomer (LogI(q) vs. q) with the GNOM fit superimposed to the scatter curve.(**B**) R_g normalized kratky plot of the retinoschisin monomer $((I(q)/I(0))(qR_g)^2$ vs. $qR_g)$ showing a peak consistent with a folded protein. Shown are the cross-hairs for globularity of the protein fold (1.1 vs. $\sqrt{3}$). This analysis suggests a folded, elongated structure. (**C**) Guinier plot (LogI(q) vs. q^2) of the low q region, showing a Radius of Gyration (R_g) of 31.6Å for the retinoschisin monomer with plotted residuals for the guinier region.

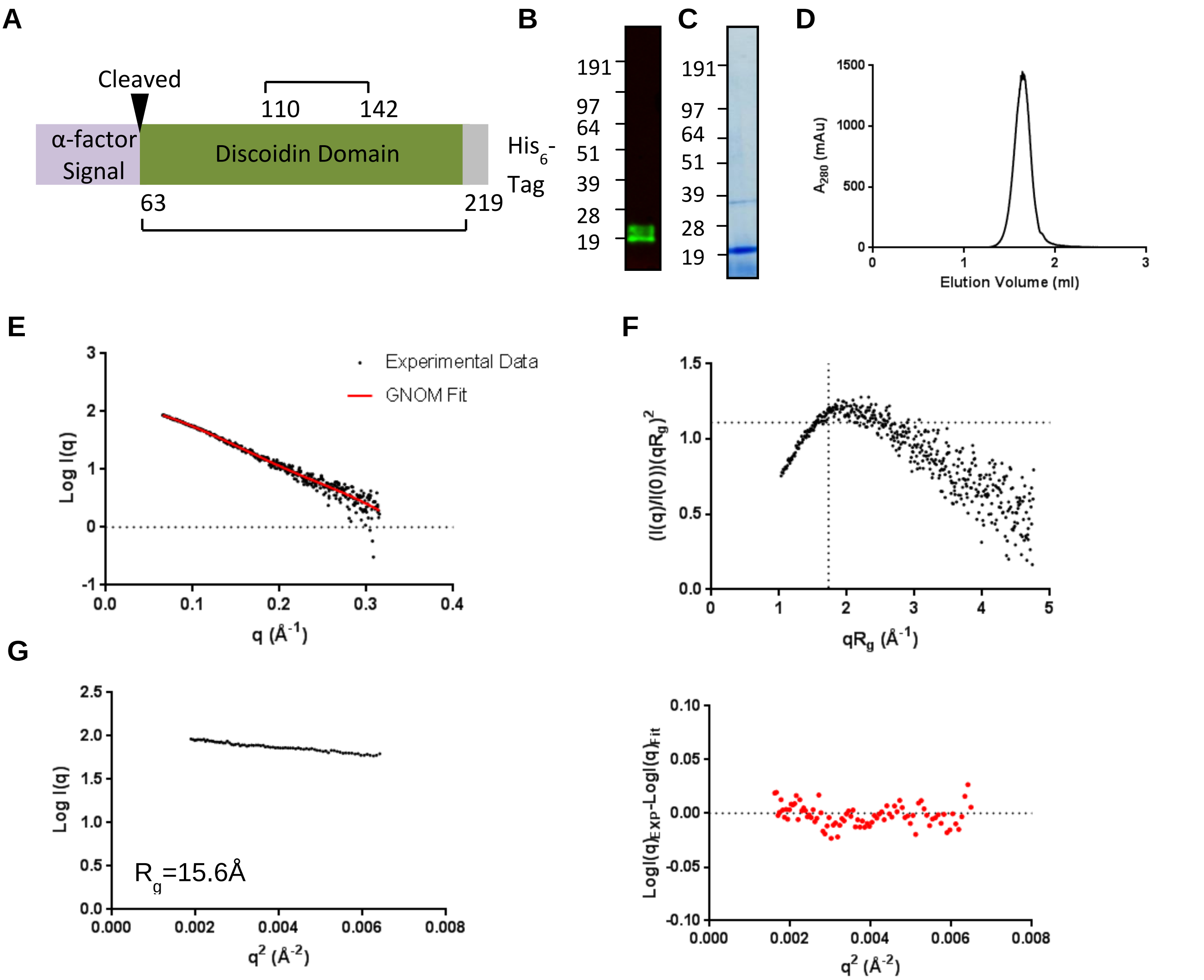

Supplementary Figure 3 – Purification of mammalian discoidin domain from *Pichia pastoris*. (**A**) Schematic diagram of the construct expressed and secreted by *Pichia pastoris.* (**B**) Western blot of the elute from Ni-NTA chromatography probed with anti-His_6 antibody (**C**) Coomassie blue stained SDS-PAGE of size-exclusion chromatography elution. (**D**) A_{280} profile of the discoidin domain from the SEC-SAXS experiment. (**E**) The rotationally averaged scatter profile of the discoidin domain (LogI(q) vs. q) with the GNOM fit superimposed to the scatter curve. (**F**) R_g normalized kratky plot of the discoidin domain $((I(q)/I(0))(qR_g)^2$ vs. $qR_g)$ showing a peak consistent with a folded protein. Shown are the cross-hairs for globularity of the protein fold (1.1 vs. $\sqrt{3}$). (**G**) Guinier plot (LogI(q) vs. q^2) of the low q region, showing R_g of 15.6Å for the discoidin domain with plotted residuals for the guinier region.

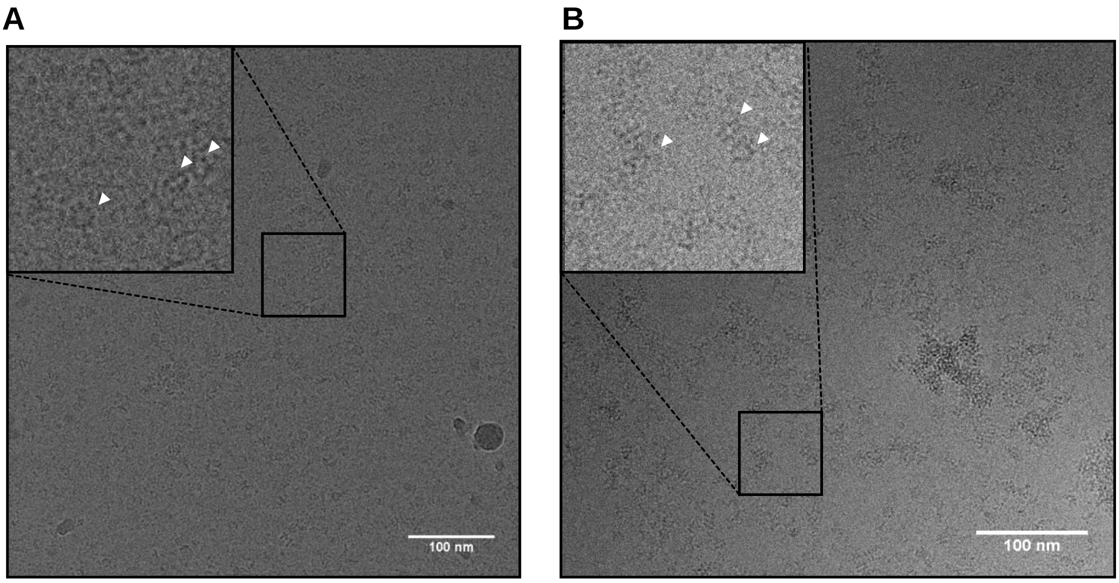

Supplementary Figure 4 – Cryo-electron microscopy of retinoschisin. (**A**) Representative cryo-EM image with a field of dispersed particles for wild-type and (**B**) R141H octamers.

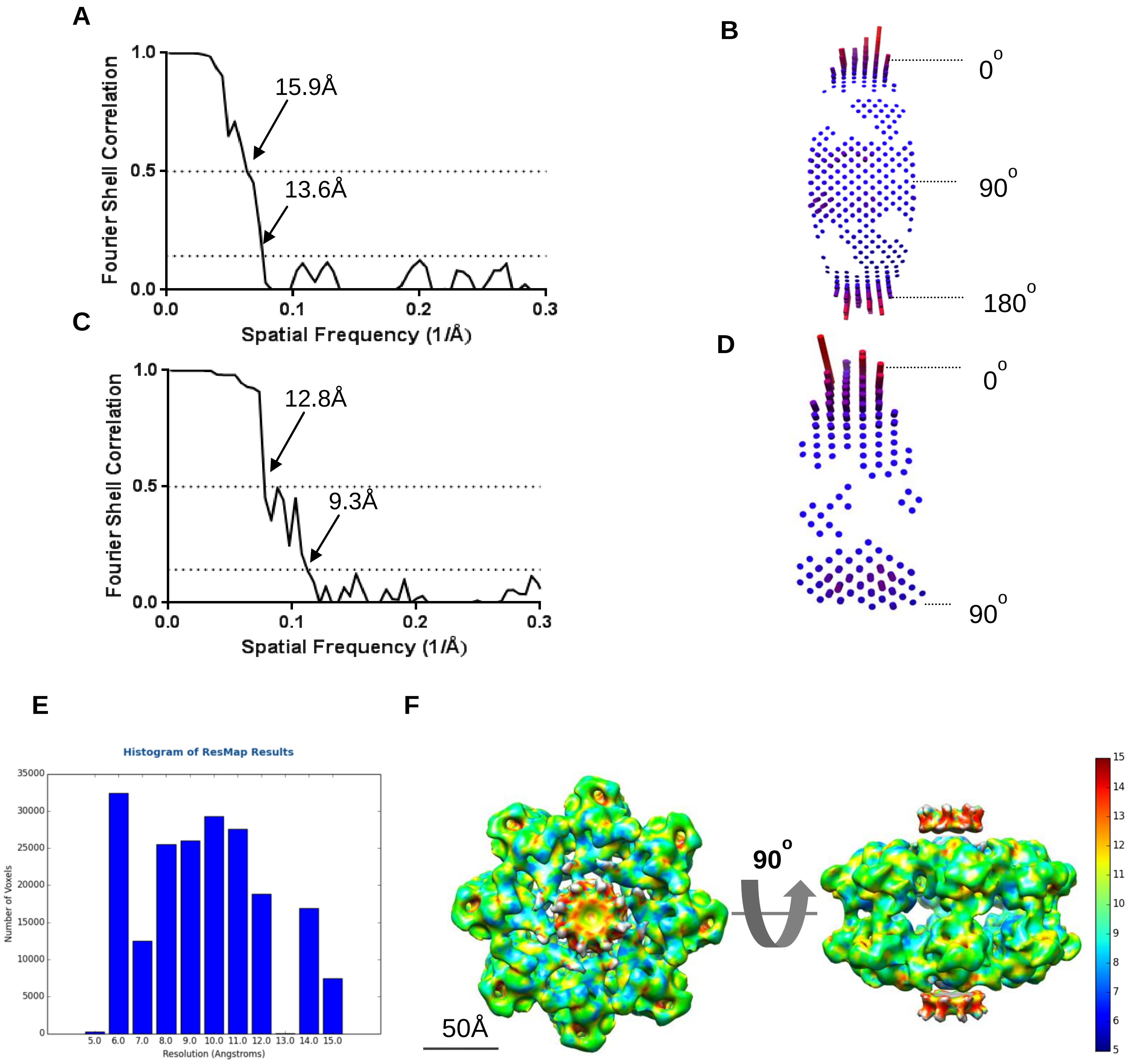

Supplementary Figure 5 – Resolution estimation of the wild-type octamer and dimer of octamers maps. (**A**) Final Fourier shell correlation (FSC) of the RELION-refined octamer map. Shown are resolution estimates at 0.5 and 0.143 correlations. (**B**) Euler angle distribution of the octamer 3D reconstruction. (**C**) Final FSC of the RELION-refined dimer of octamers map. Shown are resolution estimates at 0.5 and 0.143 correlations. (**D**) Euler angle distribution of the dimer of octamers 3D reconstruction. (**E**) ResMap-H2 analysis of the dimer of octamers map, showing the relative proportions of voxels observed at different resolutions. (**F**) ResMap-H2 analysis visualised on wild-type dimer of octamers structure, showing local resolution variations throughout the map.

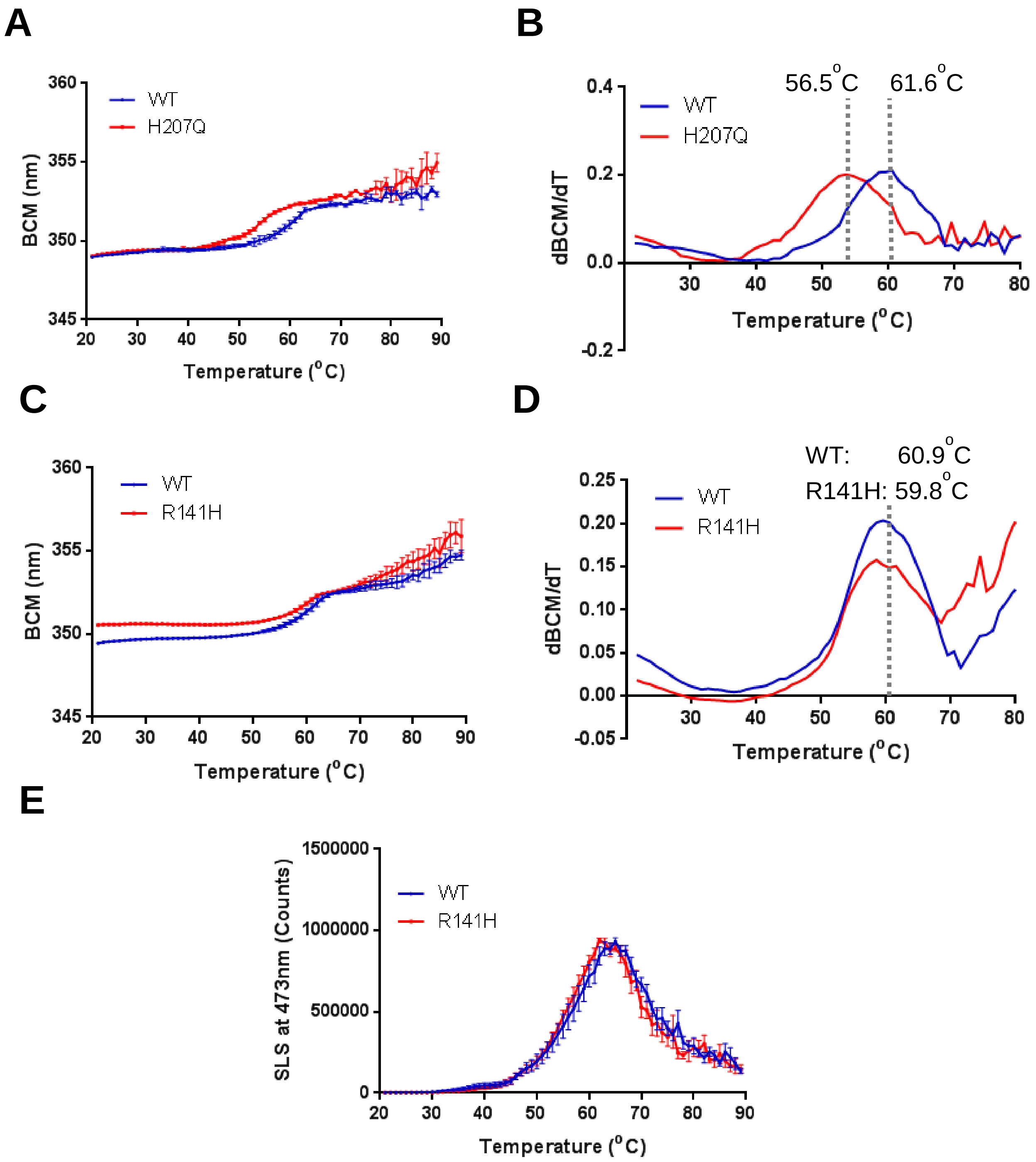

Supplementary Figure 6 – Comparative thermal stability of wild-type, R141H and H207Q retinoschisin. (**A**) Barycentric Mean Intrinsic Fluorescence Measurements of wild-type and H207Q retinoschisin monomer across a temperature range of 20-90°C, with the differentials of the temperature ramp shown in (**B**), giving the T_m for both species. (**C**) Barycentric Mean Intrinsic Fluorescence Measurements of wild-type and R141H retinoschisin monomer across a temperature range of 20-90°C, with the differentials of the temperature ramp shown in (**D**), giving similar T_m for both species. (**E**) Static Light Scattering at 473nm for wild-type and R141H monomers across a temperature range of 20 – 90 °C showing similar onset of aggregation for both species.

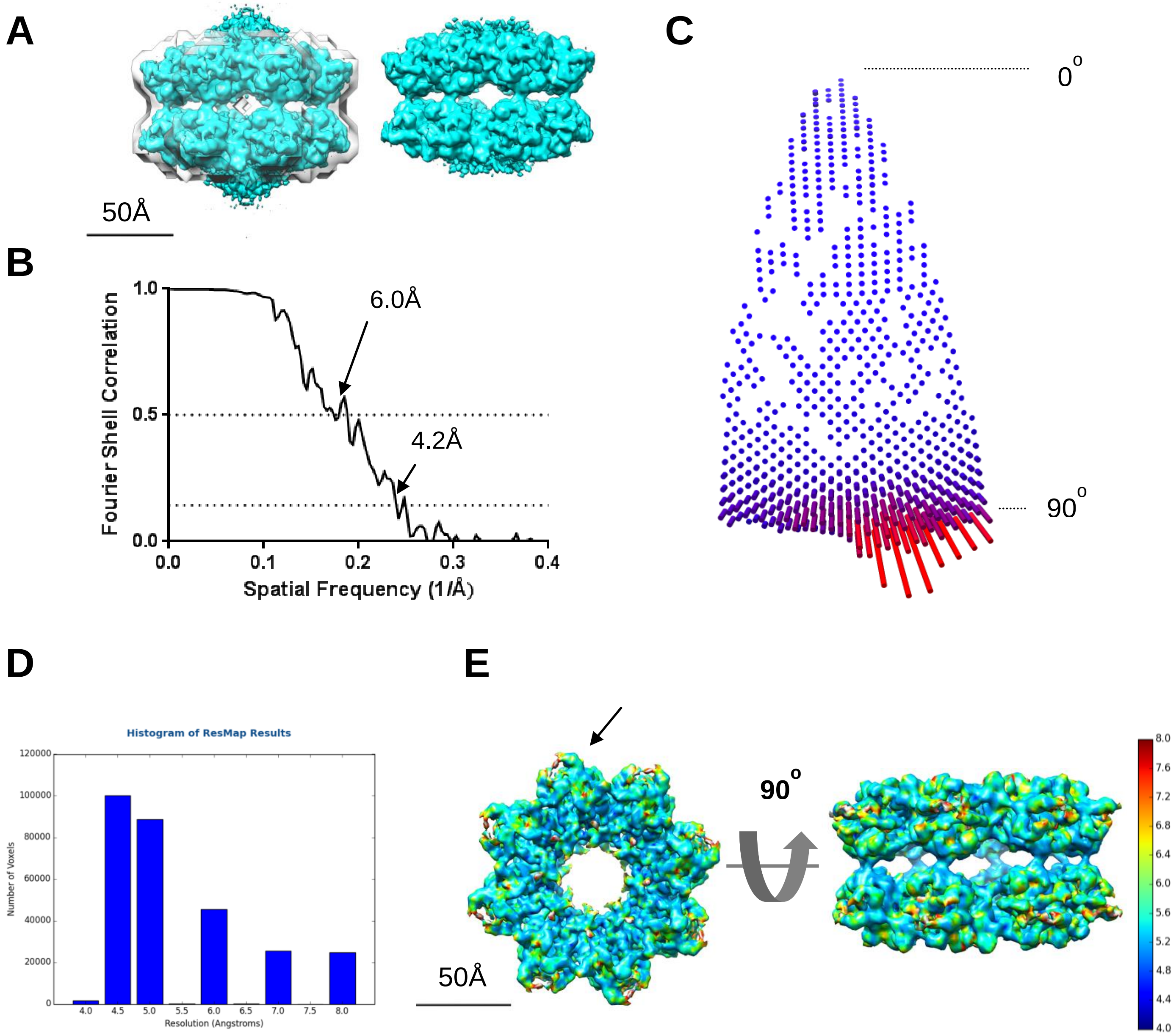

Supplementary Figure 7 – Resolution estimation of the R141H dimer of octamers structure. (**A**) The application of the soft Gaussian mask to the retinoschisin dimer of octamers and the resulting masked structure used for domain fitting and resolution estimation is shown. (**B**) Final FSC of the masked RELION-refined map. Shown are resolution estimates at 0.5 and 0.143 correlations. (**C**) Euler angle distribution of the 3D reconstruction. (**D**) ResMap-H2 analysis of the R141H map, showing the relative proportions of voxels observed at different resolutions. (**E**) ResMap-H2 analysis visualised on R141H structure, showing local resolution variations throughout the map, regions with reduced resolution are marked with arrows.

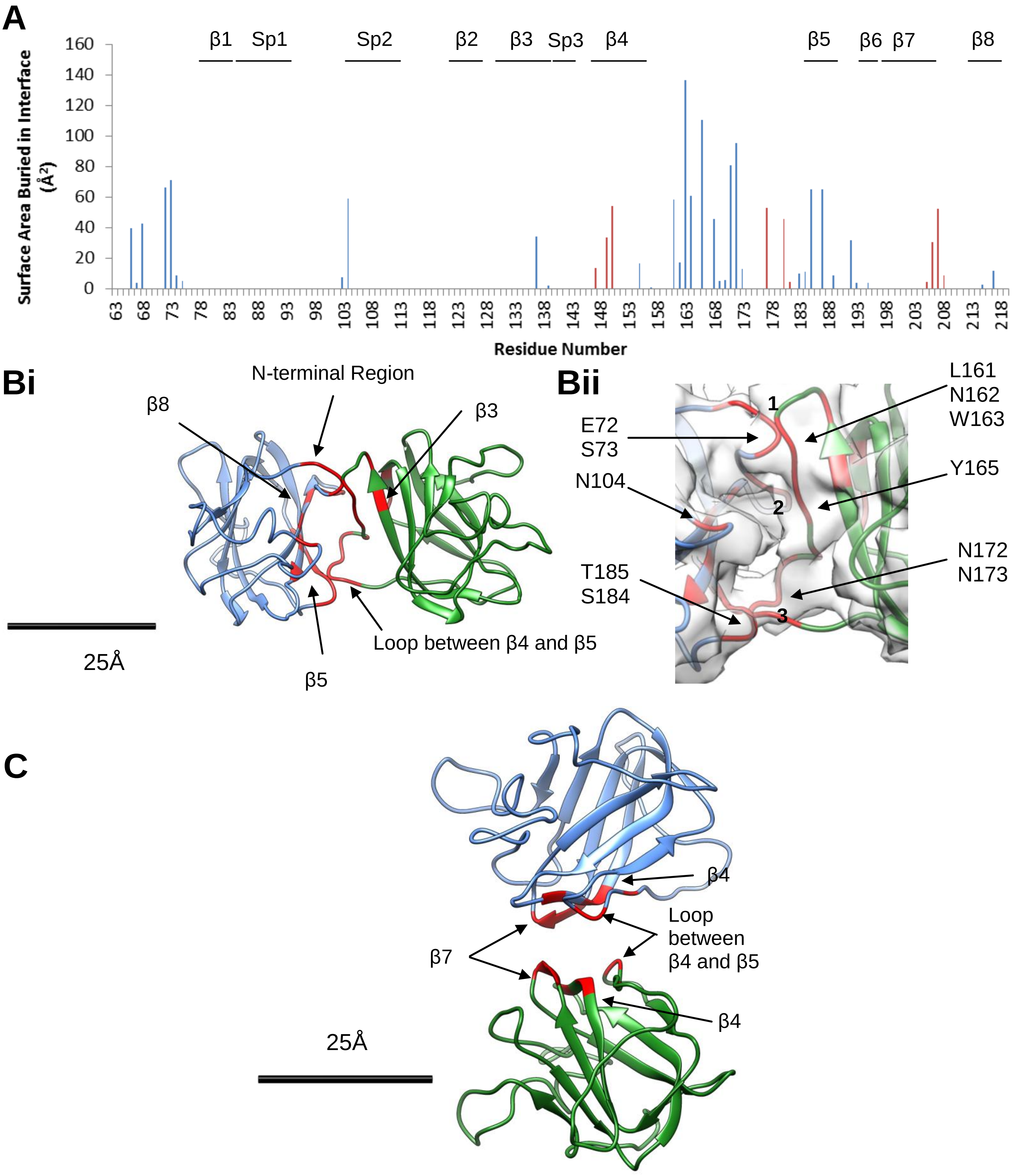

Supplementary Figure 8 – Mapping of discoidin domain interfaces in the quasi-atomic model. (**A**) PDBePISA analysis of the interfaces within the hexadecamer. Residues which contribute to the intra-octamer interface are shown in blue and residues involved in the inter-octamer interface highlighted in red. (**Bi**) The intra-octamer interface between subunits within the octamer is shown with residues identified by PDBePISA highlighted. (**ii**) The intra-octamer interface contains 3 main contact points, with residues in these regions labelled. (**C**) The inter-octamer interface is shown with PDBePISA-identified regions shown in red.

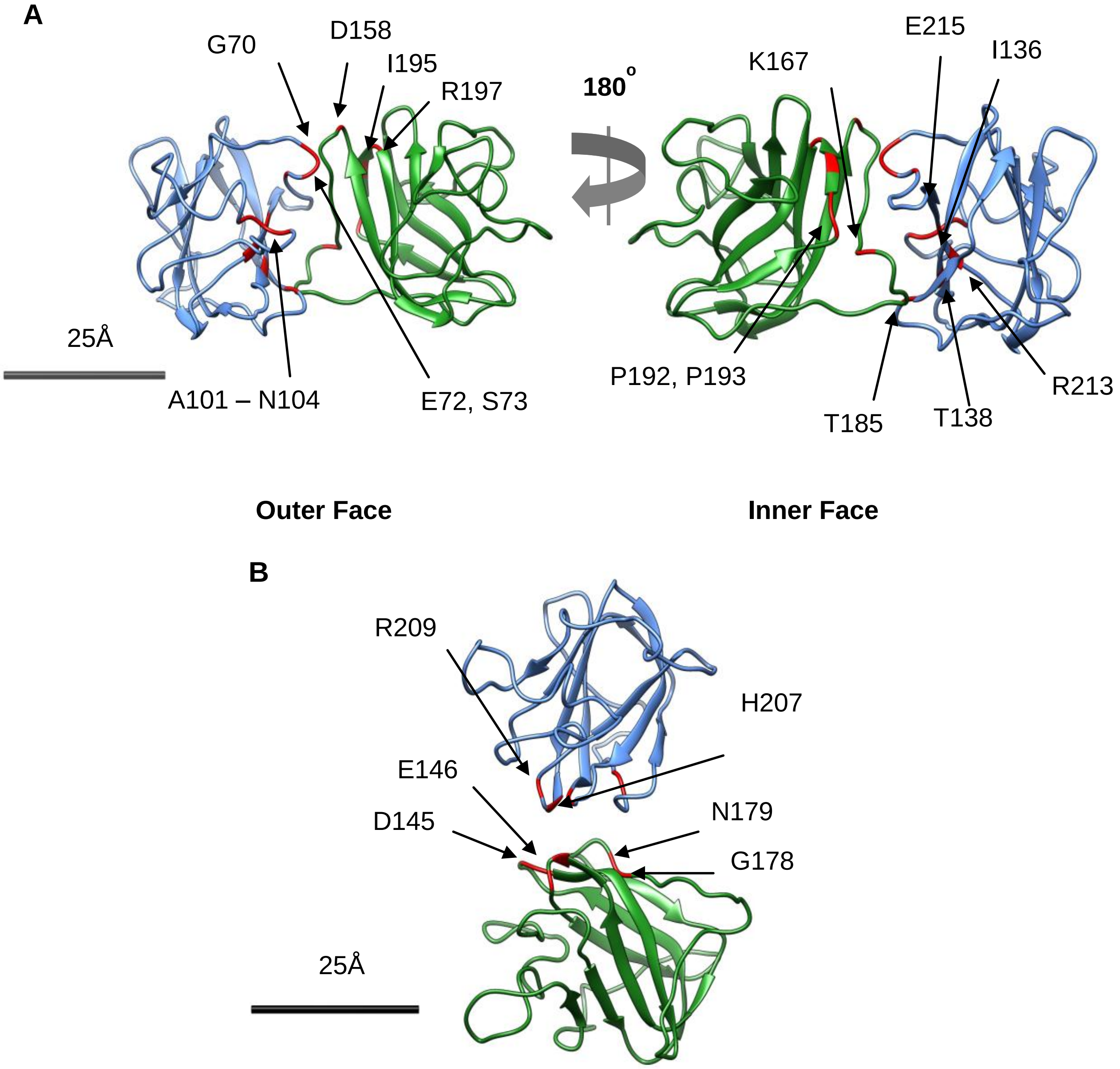

Supplementary Figure 9 - Mapping XLRS-causative conservative mutations onto the quasi-atomic model. (**A**) The positions of non-cysteine, conservative XLRS-causative mutations which map to the intra-octamer interface are displayed in red. Shown are the mutations in the interface from both the outer and inner faces of the discoidin domain ring structure within the octamer. (**B**) Non-cysteine conservative XLRS-causative mutations, which map to the inter-octamer interface, are highlighted in red.
